# X-ray structural insights and computational analysis of the compound 5-ethyl-4-[(4-morpholino­benzylidene)amino]-2,4-di­hydro-3*H*-1,2,4-triazole-3-thione

**DOI:** 10.1107/S2056989025004852

**Published:** 2025-06-03

**Authors:** Syed Nizamuddin, T. N. Mahadeva Prasad, N. R. Sreenatha, C. L. Sharath, B. N. Lakshminarayana, K. A. Vishnumurthy

**Affiliations:** a Department of PG Studies and Research in Industrial Chemistry Kuvempu University, Shivamogga Karnataka India; b Department of Chemistry, Adichunchanagiri Institute of Technology, Chikkamagaluru 577102, Karnataka, India; c Department of Physics, Government First Grade College, Gundlupet 571111, Karnataka, India; d Department of Physics, Government Engineering College, Chamarajanagara 571313, Karnataka, India; e Department of Studies in Chemistry Mangalore University, Mangalagangothri Mangaluru 574199 Karnataka India; f Department of Physics, Adichunchanagiri Institute of Technology, Chikkamagaluru 577102, Karnataka, India; g Retired Joint Director Department of Collegiate Education Government of Karnataka, Regional Office Shivamogga India; University of Buenos Aires, Argentina

**Keywords:** single crystal XRD, chair conformation, Hirshfeld surfaces, 2D fingerprint plots

## Abstract

The title compound, C_15_H_19_N_5_OS, crystallizes in the monoclinic crystal system, space group *P*2_1_/*c*. The mol­ecule adopts a non-planar geometry. A significant feature of the structure is the puckered six-membered morpholine ring, which adopts a chair conformation. In the crystal, mol­ecules are linked through inter­molecular N—H⋯S hydrogen bonds, forming inversion-related dimers with an *R*^2^_2_(8) ring motif. A Hirshfeld surface analysis was undertaken to qu­antify the inter­molecular inter­actions that influence the crystal packing.

## Abstract 2

The mol­ecule of the title compound adopts a non-planar geometry. A significant feature is the puckered six-membered morpholine ring, which adopts a chair conformation. In the crystal, mol­ecules are linked through inter­molecular N—H⋯S hydrogen bonds, forming inversion-related dimers with an *R*^2^_2_(8) ring motif.

## 1. Chemical context

Heterocyclic compounds play a vital role in pharmaceutical research due to their wide range of biological activities. The present compound contains both a six-membered morpholine ring and a five-membered triazole moiety, each known for their therapeutic potential. Morpholine derivatives are also valued in industrial applications, such as corrosion inhibition in shale gas pipelines, owing to their low toxicity and anti­micro­bial properties (Wang *et al.*, 2024 ▸; Zhao *et al.*, 2024 ▸). Beyond pharmaceuticals, morpholine has gained attention for its use in fruit wax coatings, where its potential conversion to carcinogenic *N*-nitroso­morpholine highlights significant health risks and regulatory importance (Sundarrajan *et al.*, 2025 ▸). Additionally, *N*-hetero­aryl­morpholine frameworks are frequently found in drugs used to treat conditions such as schizo­phrenia and type-2 diabetes mellitus (Bandaru *et al.*, 2018 ▸).
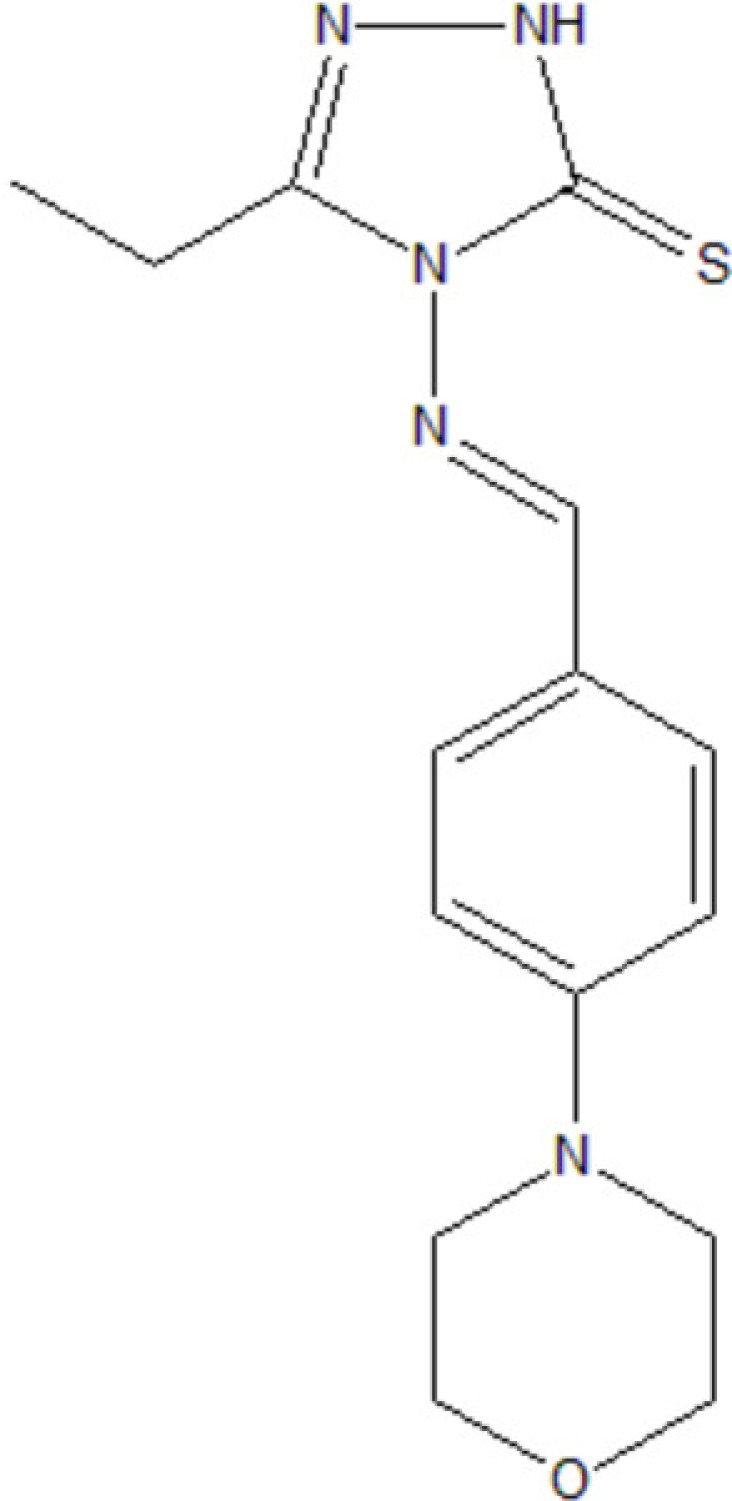


In the light of these diverse applications and biological significance, we report the structural and computational analysis of the compound 5-ethyl-4-[(4-morpholino­benzyl­idene)amino]-2,4-di­hydro-3H-1,2,4-triazole-3-thione.

## 2. Structural commentary

**Figure 1:**
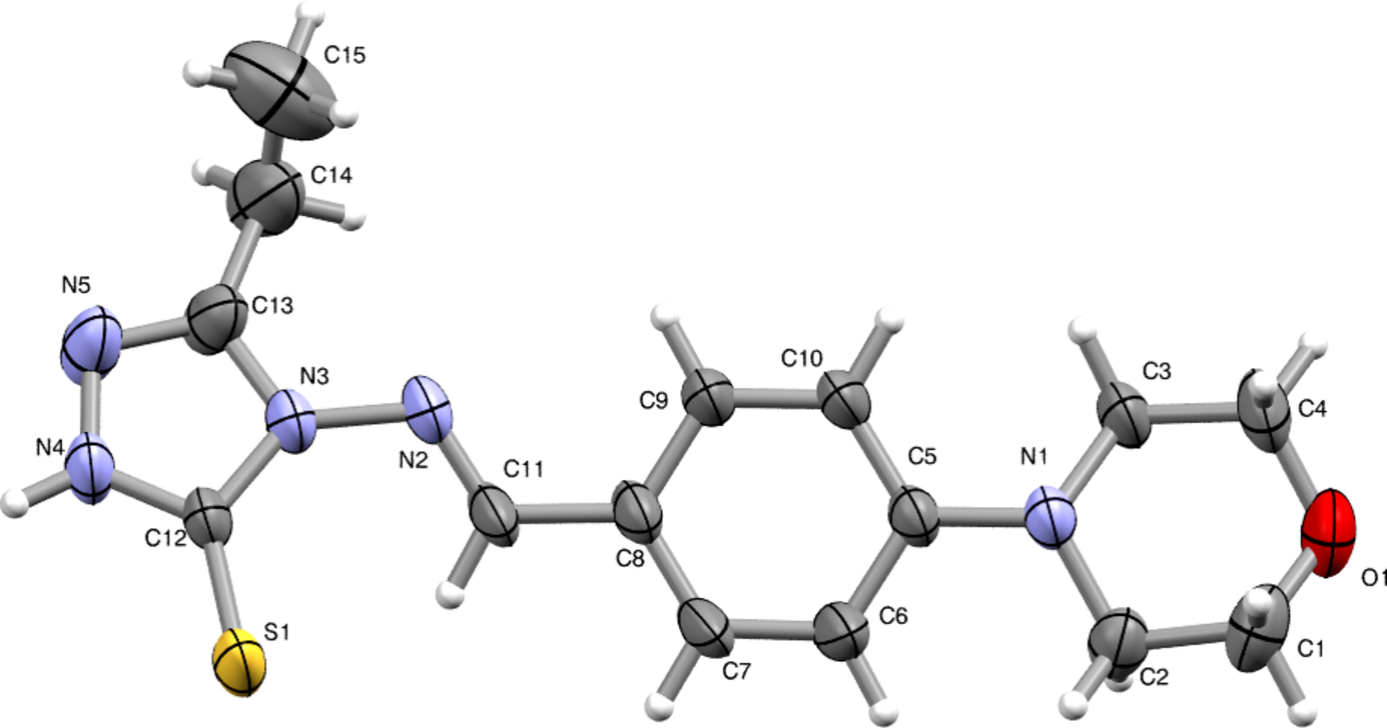
The mol­ecular structure of the title compound with displacement ellipsoids drawn at the 50% probability level.

The mol­ecular structure is illustrated in Fig. 1 ▸. The mol­ecule exhibits a slightly non-planar geometry. The dihedral angle between the mean planes of the morpholine ring (C1–C2–N1–C3–C4–O1) and the triazole ring (N3–C12–N4–N5–C13) is 11.42 (2)°, indicating a twisted conformation across the central phenyl ring (C5–C10). The morpholine ring adopts a chair conformation with puckering amplitude *Q* = 0.545 (3)Å, θ = 175.1 (3)° and relative phase angle of 177 (4)°. The ethyl side chain at C13 adopts a +syn-clinal orientation, as indicated by a N5—C13—C14—C15 torsion angle of 88.9 (5)°. The sulfur atom at C12 is in a +anti-periplanar arrangement with respect to the triazole ring, with a torsion angle of 176.5 (2)° for the chain of N5—N4—C12—S1 atoms. Bond lengths and angles are in good agreement with those in reported structures (Lakshminarayana *et al.*, 2022 ▸; Di Salvo *et al.*, 2011 ▸; Sreenatha *et al.*, 2017 ▸).

## 3. Supra­molecular features

**Figure 2:**
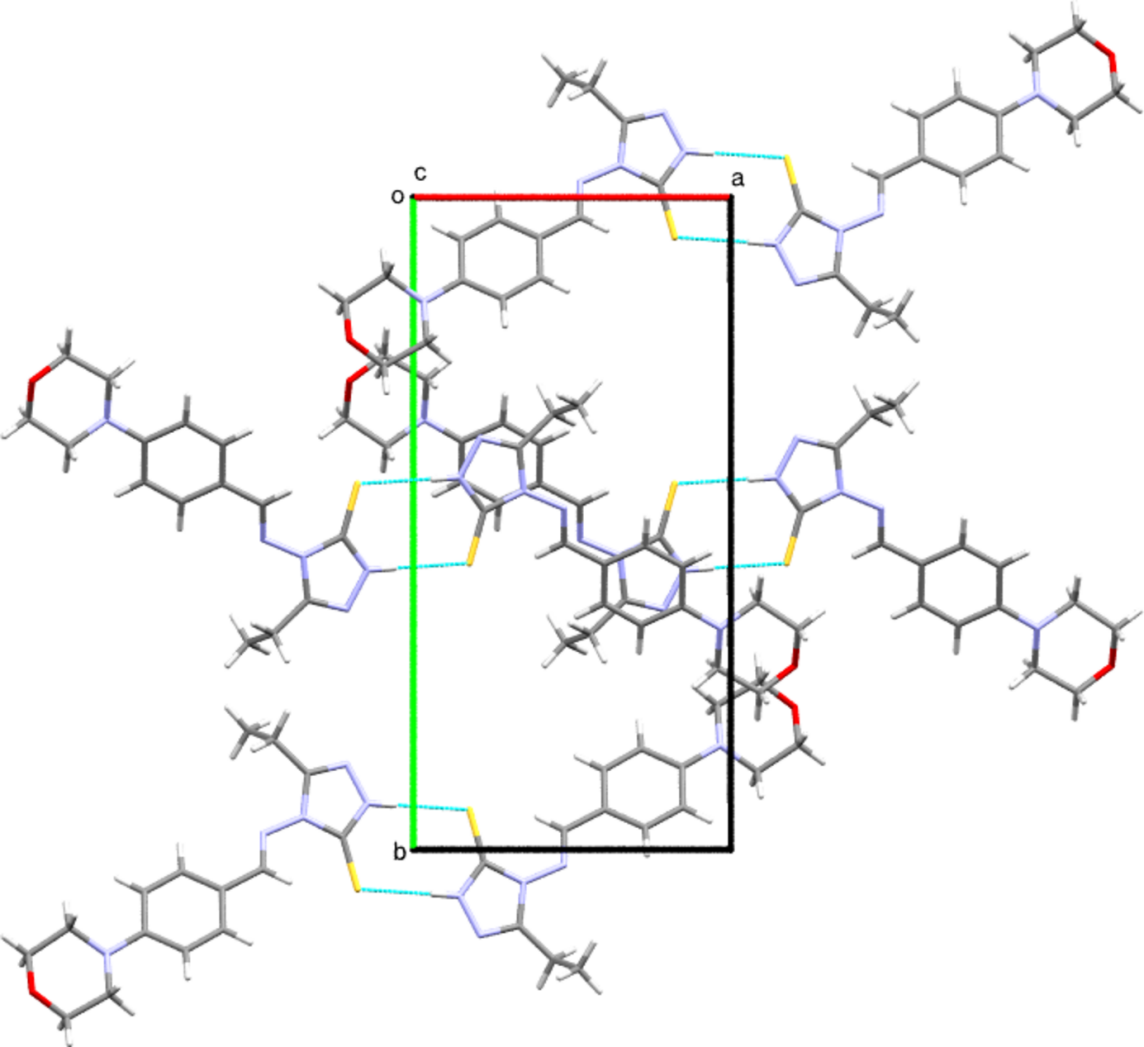
The packing of title mol­ecules *via* inter­molecular N4—H4*N*⋯S1 inter­actions, viewed along the crystallographic *b*-axis direction.

**Figure 3:**
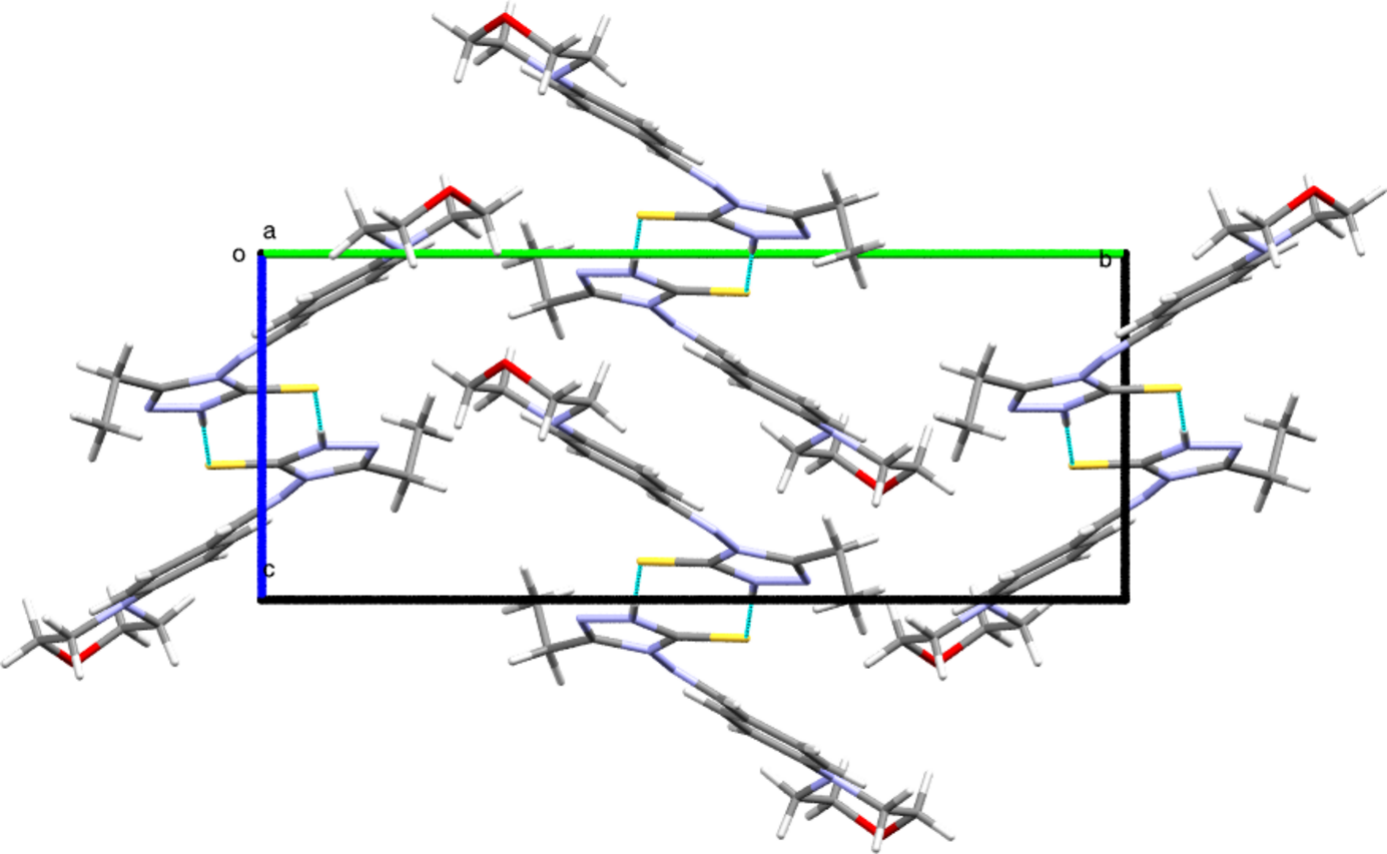
The packing viewed along the crystallographic *a-*axis direction.

In the crystal, mol­ecules are connected by inter­molecular N4—H4*N*⋯S1 hydrogen bonds (Table 1 ▸), forming inversion dimers characterized by an 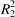
(8) motif. The two-dimensional projection along the crystallographic *b*-axis direction is shown in Fig. 2 ▸. The packing mode along the crystallographic a-axis is shown in Fig. 3 ▸.

## 4. Database survey

A search of the Cambridge Structural Database (CSD,2025version; Groom *et al.*, 2016 ▸) for morpholine-containing compounds yielded numerous hits. Among them, AHEPUY (Oswald *et al.*, 2002 ▸) shows paracetamol mol­ecules hydrogen-bonded *via* N—H⋯O and C=O⋯H inter­actions mediated by morpholine. The morpholine derivative AGAZAL (Sarbu *et al.*, 2013 ▸) exhibits O—H⋯O, C—H⋯O,and C—H⋯S inter­actions, supporting the relevance of such motifs in structural studies.

## 5. Hirshfeld surfaces and 2D fingerprint calculations

**Figure 4:**
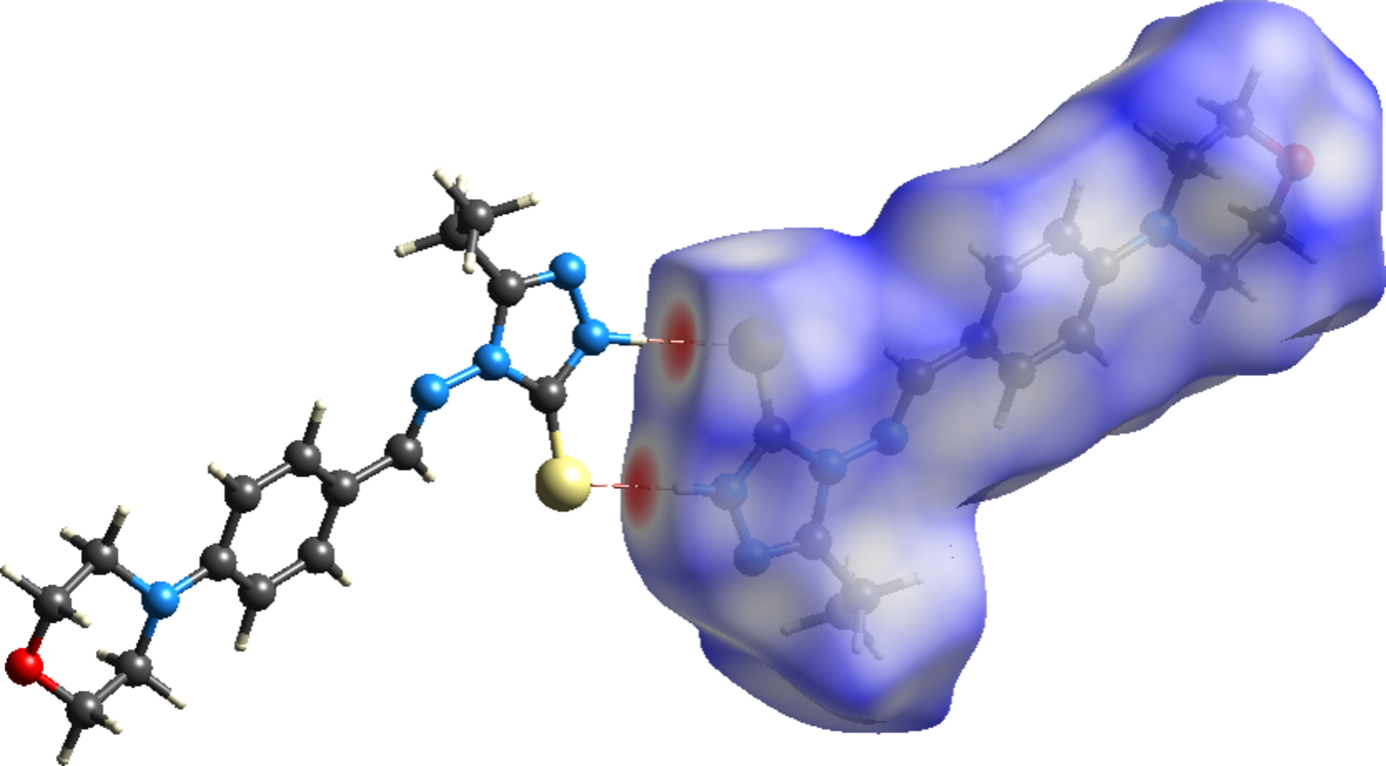
The Hirshfeld surface of the title compound mapped over *d*_norm_ with red spots corresponding to the inter­molecular N4—H4*N*⋯S1 inter­actions.

**Figure 5:**
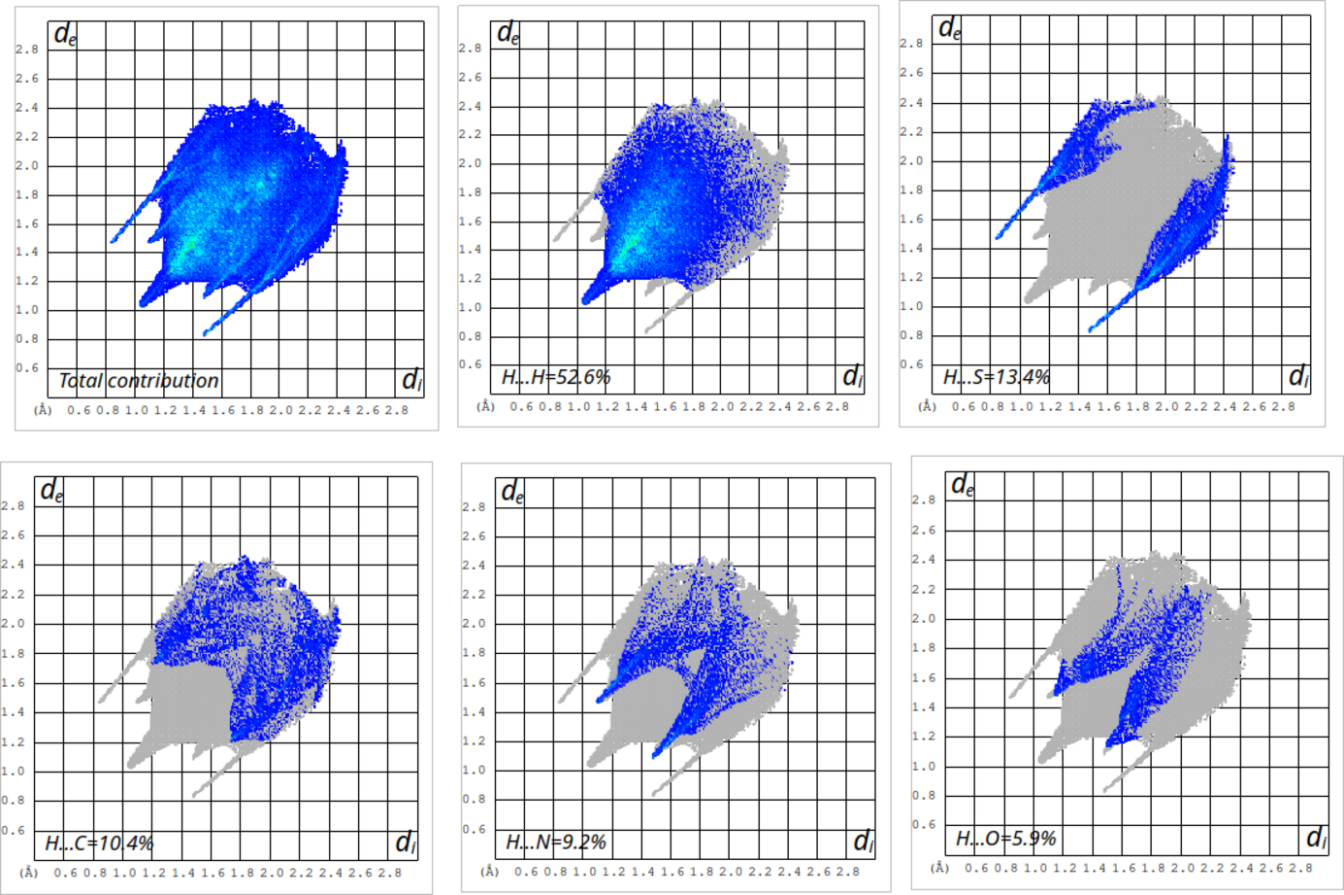
Two-dimensional fingerprint graphs showing the total contribution and those delineated into H⋯H, S⋯H/H⋯S, C⋯H/H⋯C, N⋯H/H⋯N and O⋯H/H⋯O contacts.

Hirshfeld surface analysis and corresponding fingerprint plots were generated using *CrystalExplorer* software. (Spackman *et al.*, 2021 ▸; Spackman & Jayatilaka, 2009 ▸). The surface mapped over *d*_norm_ shows red spots corresponding to regions of strong inter­molecular inter­actions (Fig. 4 ▸). The 2D fingerprint plots (Fig. 5 ▸) qu­antify the contributions of various contact types: H⋯H inter­actions contribute the most at 52.6%, forming characteristic blue ‘wings’ around 1.01 Å. The S⋯H/H⋯S inter­actions contribute 13.4%, forming a distinct ‘scorpion-pin’ motif near *d*_e_ + *d*_i_ ≃ 2.37 Å. C⋯H/H⋯C contacts account for 10.4%, forming lung-shaped patterns at *d*_e_ + *d*_i_ ≃ 2.91 Å. N⋯H/H⋯N contacts contribute 9.2%, appearing as spike-like features near *d*_e_ + *d*_i_ ≃ 2.61Å. O⋯H/H⋯O contacts contribute 5.9%, forming wing-like shapes at *d*_e_ + *d*_i_ ≃ 2.71Å

## 6. Synthesis and crystallization

**Figure 6:**
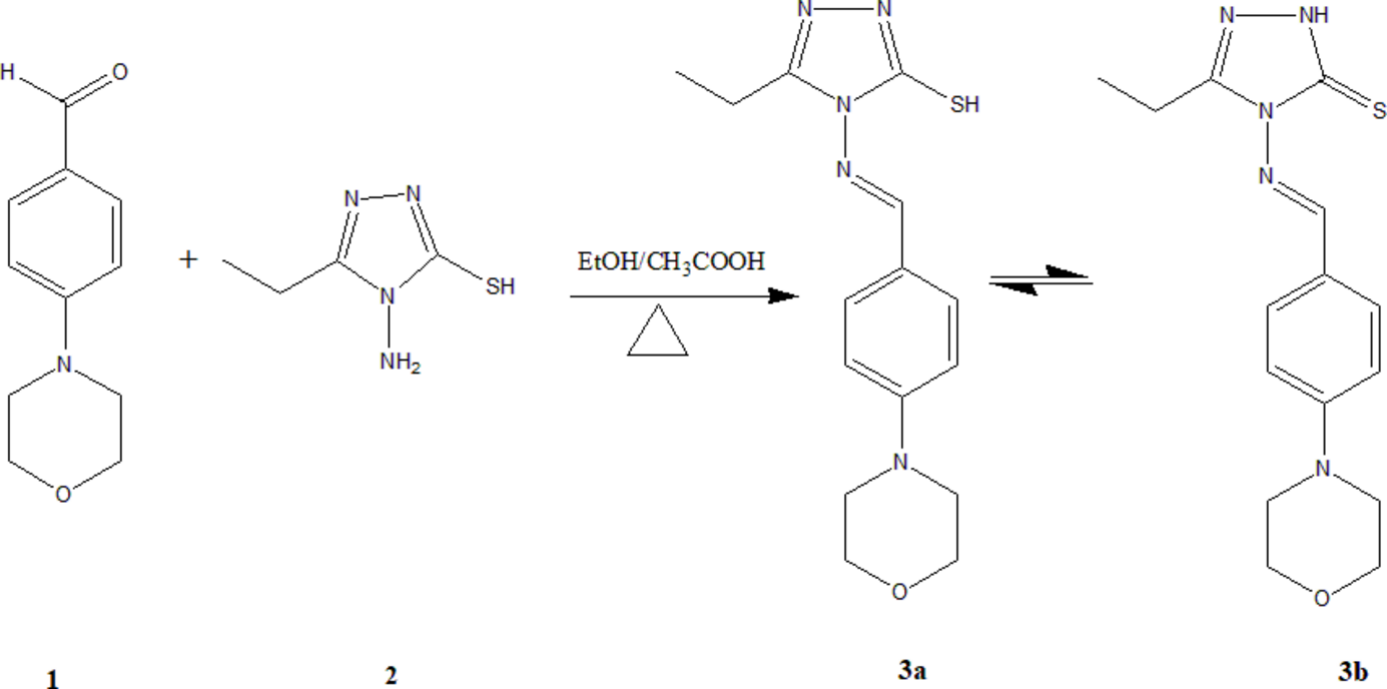
The synthesis scheme for the title compound.

An equimolar mixture of *p*-morpholino­benzaldehyde (**1**) and 4-amino-5-ethyl-4*H*-1,2,4-triazole-3-thiol (**2**) was refluxed in ethanol (10 mL) with a few drops of acetic acid for 6 h. Reaction progress was monitored by TLC. After completion, the solution was cooled to room temperature. The resulting solid was filtered, dried, and recrystallized from ethanol solution to obtain crystals suitable for X-ray analysis. A reaction scheme is provided in Fig. 6 ▸ (for more details of the synthesis, see:Dhaka *et al.*, 1974 ▸; Liu & Yan, 2008 ▸). The crystallized compound corresponds to the thione form (**3b**), which may be favored in the solid state due to possible inter­molecular N4—H4*N*⋯S1 hydrogen-bonding inter­actions in the crystal. However, similar stabilizing forces could also operate in the thiol tautomer (**3a**).

## 7. Refinement

Crystal data, data collection and structure refinement details are summarized in Table 2 ▸. C-bound hydrogen atoms were placed in idealized positions and refined as riding with C—H = 0.93 Å with *U*_iso_(H) = 1.2*U*_eq_(C). The NH atom was freely refined.

## Supplementary Material

Crystal structure: contains datablock(s) global, I. DOI: 10.1107/S2056989025004852/vu2012sup1.cif

Supporting information file. DOI: 10.1107/S2056989025004852/vu2012Isup3.cml

Structure factors: contains datablock(s) I. DOI: 10.1107/S2056989025004852/vu2012Isup3.hkl

CCDC reference: 2312837

Additional supporting information:  crystallographic information; 3D view; checkCIF report

## Figures and Tables

**Table 1:** Hydrogen-bond geometry (Å, °)

*D*—H⋯*A*	*D*—H	H⋯*A*	*D*⋯*A*	*D*—H⋯*A*
N4—H4*N*⋯S1^i^	0.87 (4)	2.474 (4)	3.335 (2)	179 (14)

Symmetry code: (i) 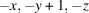.

**Table 2:** Experimental details

Crystal data	
Chemical formula	C_15_H_19_N_5_OS
*M*_r_	317.41
Crystal system, space group	Monoclinic, *P*2_1_/*c*
Temperature (K)	296
*a*, *b*, *c* (Å)	9.7280 (8), 19.9593 (15), 8.0087 (6)
β (°)	93.230 (4)
*V* (Å^3^)	1552.5 (2)
*Z*	4
Radiation type	Mo *K*α
μ (mm^−1^)	0.22
Crystal size (mm)	0.80 × 0.70 × 0.60

Data collection	
Diffractometer	Bruker SMART APEX
No. of measured, independent and observed [*I* > 2σ(*I*)] reflections	16741, 3824, 2528
*R*_int_	0.043
(sin θ/λ)_max_ (Å^−1^)	0.670

Refinement	
*R*[*F*^2^ > 2σ(*F*^2^)], *wR*(*F*^2^), *S*	0.063, 0.193, 1.01
No. of reflections	3824
No. of parameters	204
H-atom treatment	H atoms treated by a mixture of independent and constrained refinement
Δρ_max_, Δρ_min_ (e Å^−3^)	0.71, −0.55

Computer programs: *APEX* and *SAINT* (Bruker, 2006 ▸), *SHELXT2014/4* (Sheldrick, 2015*a* ▸), *SHELXL2019/2* (Sheldrick, 2015*b* ▸) and *PLATON*(Spek, 2020 ▸).

## supplementary crystallographic information

### 5-Ethyl-4-[(4-morpholinobenzylidene)amino]-2,4-dihydro-3H-1,2,4-triazole-3-thione Crystal data

**Table:**

C_15_H_19_N_5_OS	*F*(000) = 672
*M**_r_* = 317.41	*D*_x_ = 1.358 Mg m^−^^3^
Monoclinic, *P*2_1_/*c*	Mo *K*α radiation, λ = 0.71073 Å
*a* = 9.7280 (8) Å	Cell parameters from 3824 reflections
*b* = 19.9593 (15) Å	θ = 2.3–28.4°
*c* = 8.0087 (6) Å	µ = 0.22 mm^−^^1^
β = 93.230 (4)°	*T* = 296 K
*V* = 1552.5 (2) Å^3^	Block, colorless
*Z* = 4	0.80 × 0.70 × 0.60 mm

### 5-Ethyl-4-[(4-morpholinobenzylidene)amino]-2,4-dihydro-3H-1,2,4-triazole-3-thione Data collection

**Table:**

Bruker SMART APEX diffractometer	*R*_int_ = 0.043
Radiation source: graphite	θ_max_ = 28.4°, θ_min_ = 2.3°
Detector resolution: 0.812 pixels mm^-1^	*h* = −12→13
16741 measured reflections	*k* = −26→26
3824 independent reflections	*l* = −10→10
2528 reflections with *I* > 2σ(*I*)	

### 5-Ethyl-4-[(4-morpholinobenzylidene)amino]-2,4-dihydro-3H-1,2,4-triazole-3-thione Refinement

**Table:**

Refinement on *F*^2^	0 restraints
Least-squares matrix: full	Hydrogen site location: mixed
*R*[*F*^2^ > 2σ(*F*^2^)] = 0.063	H atoms treated by a mixture of independent and constrained refinement
*wR*(*F*^2^) = 0.193	*w* = 1/[σ^2^(*F*_o_^2^) + (0.091*P*)^2^ + 1.1496*P*] where *P* = (*F*_o_^2^ + 2*F*_c_^2^)/3
*S* = 1.01	(Δ/σ)_max_ < 0.001
3824 reflections	Δρ_max_ = 0.71 e Å^−^^3^
204 parameters	Δρ_min_ = −0.55 e Å^−^^3^

### 5-Ethyl-4-[(4-morpholinobenzylidene)amino]-2,4-dihydro-3H-1,2,4-triazole-3-thione Special details

**Table:**

Geometry. All esds (except the esd in the dihedral angle between two l.s. planes) are estimated using the full covariance matrix. The cell esds are taken into account individually in the estimation of esds in distances, angles and torsion angles; correlations between esds in cell parameters are only used when they are defined by crystal symmetry. An approximate (isotropic) treatment of cell esds is used for estimating esds involving l.s. planes.

### 5-Ethyl-4-[(4-morpholinobenzylidene)amino]-2,4-dihydro-3H-1,2,4-triazole-3-thione Fractional atomic coordinates and isotropic or equivalent isotropic displacement parameters (Å^2^)

**Table:**

	*x*	*y*	*z*	*U*_iso_*/*U*_eq_	
C1	1.0926 (3)	0.75825 (16)	0.6166 (4)	0.0617 (8)	
H1A	1.113183	0.772682	0.505069	0.074*	
H1B	1.084745	0.797943	0.685299	0.074*	
C2	0.9581 (3)	0.72168 (13)	0.6080 (4)	0.0505 (7)	
H2A	0.932333	0.711318	0.720376	0.061*	
H2B	0.887212	0.750150	0.555856	0.061*	
C3	1.0849 (2)	0.61946 (14)	0.5713 (4)	0.0517 (7)	
H3A	1.095855	0.582320	0.495109	0.062*	
H3B	1.068877	0.601234	0.680808	0.062*	
C4	1.2142 (3)	0.66126 (18)	0.5813 (4)	0.0648 (9)	
H4A	1.290469	0.634002	0.625262	0.078*	
H4B	1.234677	0.675563	0.469684	0.078*	
C5	0.8470 (2)	0.62665 (12)	0.4614 (3)	0.0388 (5)	
C6	0.7183 (3)	0.65485 (17)	0.4808 (4)	0.0639 (9)	
H6	0.711682	0.696074	0.533834	0.077*	
C7	0.6005 (3)	0.62238 (18)	0.4223 (4)	0.0666 (10)	
H7	0.515733	0.642494	0.436839	0.080*	
C8	0.6032 (2)	0.56162 (14)	0.3435 (3)	0.0431 (6)	
C9	0.7306 (2)	0.53358 (13)	0.3225 (4)	0.0452 (6)	
H9	0.736421	0.492724	0.267475	0.054*	
C10	0.8490 (2)	0.56506 (13)	0.3814 (4)	0.0497 (7)	
H10	0.933271	0.544418	0.367359	0.060*	
C11	0.4752 (2)	0.52978 (15)	0.2829 (3)	0.0490 (7)	
H11	0.391832	0.549583	0.305837	0.059*	
C12	0.2230 (2)	0.48038 (13)	0.1063 (3)	0.0410 (6)	
C13	0.3334 (3)	0.38280 (15)	0.1116 (4)	0.0602 (8)	
C14	0.4417 (4)	0.3313 (2)	0.1434 (5)	0.0869 (13)	
H14A	0.503355	0.346216	0.235420	0.104*	
H14B	0.398600	0.290136	0.177690	0.104*	
C15	0.5207 (5)	0.3176 (3)	0.0030 (7)	0.1182 (18)	
H15A	0.460516	0.303478	−0.089486	0.177*	
H15B	0.585800	0.282663	0.031072	0.177*	
H15C	0.568966	0.357333	−0.027256	0.177*	
N1	0.9671 (2)	0.66008 (10)	0.5129 (3)	0.0427 (5)	
N2	0.4746 (2)	0.47624 (12)	0.2003 (3)	0.0460 (5)	
N3	0.3474 (2)	0.44995 (11)	0.1461 (3)	0.0439 (5)	
N4	0.1450 (2)	0.42896 (11)	0.0544 (3)	0.0497 (6)	
N5	0.2104 (3)	0.36884 (12)	0.0545 (4)	0.0646 (7)	
O1	1.2022 (2)	0.71815 (11)	0.6838 (3)	0.0642 (6)	
S1	0.17662 (7)	0.56092 (4)	0.11137 (12)	0.0624 (3)	
H4N	0.061 (4)	0.4319 (17)	0.012 (4)	0.074 (11)*	

### 5-Ethyl-4-[(4-morpholinobenzylidene)amino]-2,4-dihydro-3H-1,2,4-triazole-3-thione Atomic displacement parameters (Å^2^)

**Table:**

	*U*^11^	*U*^22^	*U*^33^	*U*^12^	*U*^13^	*U*^23^
C1	0.073 (2)	0.0510 (16)	0.0588 (18)	−0.0207 (15)	−0.0122 (15)	−0.0001 (14)
C2	0.0502 (15)	0.0419 (14)	0.0578 (16)	−0.0028 (12)	−0.0111 (12)	−0.0004 (12)
C3	0.0281 (12)	0.0526 (15)	0.0730 (18)	0.0009 (11)	−0.0091 (12)	−0.0053 (14)
C4	0.0321 (13)	0.083 (2)	0.078 (2)	−0.0113 (14)	−0.0091 (13)	−0.0072 (17)
C5	0.0269 (11)	0.0447 (13)	0.0439 (13)	0.0000 (9)	−0.0042 (9)	0.0006 (10)
C6	0.0363 (14)	0.069 (2)	0.084 (2)	0.0110 (13)	−0.0127 (13)	−0.0383 (17)
C7	0.0249 (12)	0.090 (2)	0.084 (2)	0.0134 (13)	−0.0087 (13)	−0.0391 (19)
C8	0.0251 (11)	0.0588 (16)	0.0445 (13)	0.0011 (10)	−0.0052 (9)	−0.0064 (12)
C9	0.0295 (12)	0.0406 (13)	0.0647 (16)	0.0010 (10)	−0.0041 (11)	−0.0086 (12)
C10	0.0249 (11)	0.0463 (14)	0.0773 (19)	0.0018 (10)	−0.0011 (11)	−0.0094 (13)
C11	0.0215 (11)	0.0739 (19)	0.0510 (15)	0.0030 (11)	−0.0045 (10)	−0.0131 (14)
C12	0.0249 (10)	0.0449 (13)	0.0525 (14)	−0.0041 (9)	−0.0027 (10)	−0.0029 (11)
C13	0.0618 (18)	0.0494 (16)	0.0659 (18)	0.0105 (14)	−0.0268 (15)	−0.0133 (14)
C14	0.082 (3)	0.074 (2)	0.100 (3)	0.025 (2)	−0.042 (2)	−0.022 (2)
C15	0.088 (3)	0.133 (4)	0.135 (4)	0.035 (3)	0.021 (3)	0.032 (3)
N1	0.0295 (10)	0.0418 (11)	0.0558 (12)	−0.0025 (8)	−0.0054 (9)	−0.0015 (10)
N2	0.0245 (10)	0.0561 (13)	0.0564 (13)	−0.0006 (9)	−0.0080 (9)	−0.0049 (11)
N3	0.0294 (10)	0.0480 (12)	0.0529 (12)	0.0002 (9)	−0.0095 (9)	−0.0071 (10)
N4	0.0333 (11)	0.0439 (12)	0.0700 (15)	−0.0059 (9)	−0.0142 (10)	−0.0016 (11)
N5	0.0621 (16)	0.0446 (13)	0.0829 (18)	0.0005 (11)	−0.0334 (14)	−0.0060 (12)
O1	0.0509 (12)	0.0719 (14)	0.0674 (13)	−0.0175 (10)	−0.0176 (10)	−0.0056 (11)
S1	0.0338 (4)	0.0461 (4)	0.1046 (7)	0.0026 (3)	−0.0201 (4)	−0.0148 (4)

### 5-Ethyl-4-[(4-morpholinobenzylidene)amino]-2,4-dihydro-3H-1,2,4-triazole-3-thione Geometric parameters (Å, º)

**Table:**

C1—O1	1.415 (4)	C8—C11	1.457 (3)
C1—C2	1.496 (4)	C9—C10	1.372 (3)
C1—H1A	0.9700	C9—H9	0.9300
C1—H1B	0.9700	C10—H10	0.9300
C2—N1	1.452 (3)	C11—N2	1.256 (3)
C2—H2A	0.9700	C11—H11	0.9300
C2—H2B	0.9700	C12—N4	1.329 (3)
C3—N1	1.459 (3)	C12—N3	1.375 (3)
C3—C4	1.508 (4)	C12—S1	1.671 (3)
C3—H3A	0.9700	C13—N5	1.287 (4)
C3—H3B	0.9700	C13—N3	1.374 (4)
C4—O1	1.409 (4)	C13—C14	1.483 (4)
C4—H4A	0.9700	C14—C15	1.424 (6)
C4—H4B	0.9700	C14—H14A	0.9700
C5—C10	1.387 (4)	C14—H14B	0.9700
C5—N1	1.388 (3)	C15—H15A	0.9600
C5—C6	1.390 (4)	C15—H15B	0.9600
C6—C7	1.376 (4)	C15—H15C	0.9600
C6—H6	0.9300	N2—N3	1.391 (3)
C7—C8	1.368 (4)	N4—N5	1.358 (3)
C7—H7	0.9300	N4—H4N	0.87 (4)
C8—C9	1.379 (3)		
			
O1—C1—C2	112.3 (2)	C8—C9—H9	119.5
O1—C1—H1A	109.1	C9—C10—C5	122.1 (2)
C2—C1—H1A	109.1	C9—C10—H10	118.9
O1—C1—H1B	109.1	C5—C10—H10	118.9
C2—C1—H1B	109.1	N2—C11—C8	121.7 (2)
H1A—C1—H1B	107.9	N2—C11—H11	119.2
N1—C2—C1	111.0 (2)	C8—C11—H11	119.2
N1—C2—H2A	109.4	N4—C12—N3	102.2 (2)
C1—C2—H2A	109.4	N4—C12—S1	126.90 (19)
N1—C2—H2B	109.4	N3—C12—S1	130.90 (19)
C1—C2—H2B	109.4	N5—C13—N3	111.3 (2)
H2A—C2—H2B	108.0	N5—C13—C14	123.3 (3)
N1—C3—C4	110.3 (2)	N3—C13—C14	125.4 (3)
N1—C3—H3A	109.6	C15—C14—C13	114.1 (4)
C4—C3—H3A	109.6	C15—C14—H14A	108.7
N1—C3—H3B	109.6	C13—C14—H14A	108.7
C4—C3—H3B	109.6	C15—C14—H14B	108.7
H3A—C3—H3B	108.1	C13—C14—H14B	108.7
O1—C4—C3	112.3 (3)	H14A—C14—H14B	107.6
O1—C4—H4A	109.1	C14—C15—H15A	109.5
C3—C4—H4A	109.1	C14—C15—H15B	109.5
O1—C4—H4B	109.1	H15A—C15—H15B	109.5
C3—C4—H4B	109.1	C14—C15—H15C	109.5
H4A—C4—H4B	107.9	H15A—C15—H15C	109.5
C10—C5—N1	122.0 (2)	H15B—C15—H15C	109.5
C10—C5—C6	116.5 (2)	C5—N1—C2	119.3 (2)
N1—C5—C6	121.4 (2)	C5—N1—C3	117.4 (2)
C7—C6—C5	120.7 (3)	C2—N1—C3	111.8 (2)
C7—C6—H6	119.7	C11—N2—N3	117.6 (2)
C5—C6—H6	119.7	C13—N3—C12	107.9 (2)
C8—C7—C6	122.5 (2)	C13—N3—N2	120.5 (2)
C8—C7—H7	118.8	C12—N3—N2	131.4 (2)
C6—C7—H7	118.8	C12—N4—N5	115.0 (2)
C7—C8—C9	117.2 (2)	C12—N4—H4N	125 (2)
C7—C8—C11	120.1 (2)	N5—N4—H4N	119 (2)
C9—C8—C11	122.7 (2)	C13—N5—N4	103.6 (2)
C10—C9—C8	121.0 (2)	C4—O1—C1	108.7 (2)
C10—C9—H9	119.5		
			
O1—C1—C2—N1	−56.1 (3)	C1—C2—N1—C3	51.0 (3)
N1—C3—C4—O1	56.2 (3)	C4—C3—N1—C5	166.0 (2)
C10—C5—C6—C7	−0.1 (5)	C4—C3—N1—C2	−50.7 (3)
N1—C5—C6—C7	177.1 (3)	C8—C11—N2—N3	−179.3 (2)
C5—C6—C7—C8	0.1 (6)	N5—C13—N3—C12	−0.4 (4)
C6—C7—C8—C9	−0.7 (5)	C14—C13—N3—C12	−177.2 (3)
C6—C7—C8—C11	−179.5 (3)	N5—C13—N3—N2	−176.4 (3)
C7—C8—C9—C10	1.2 (4)	C14—C13—N3—N2	6.8 (5)
C11—C8—C9—C10	−180.0 (3)	N4—C12—N3—C13	1.0 (3)
C8—C9—C10—C5	−1.3 (5)	S1—C12—N3—C13	−176.7 (2)
N1—C5—C10—C9	−176.5 (3)	N4—C12—N3—N2	176.4 (3)
C6—C5—C10—C9	0.7 (4)	S1—C12—N3—N2	−1.3 (4)
C7—C8—C11—N2	175.2 (3)	C11—N2—N3—C13	−156.1 (3)
C9—C8—C11—N2	−3.6 (5)	C11—N2—N3—C12	29.0 (4)
N5—C13—C14—C15	88.9 (5)	N3—C12—N4—N5	−1.4 (3)
N3—C13—C14—C15	−94.5 (5)	S1—C12—N4—N5	176.5 (2)
C10—C5—N1—C2	−175.0 (2)	N3—C13—N5—N4	−0.5 (4)
C6—C5—N1—C2	8.0 (4)	C14—C13—N5—N4	176.5 (3)
C10—C5—N1—C3	−34.5 (4)	C12—N4—N5—C13	1.2 (4)
C6—C5—N1—C3	148.4 (3)	C3—C4—O1—C1	−60.1 (3)
C1—C2—N1—C5	−166.5 (2)	C2—C1—O1—C4	59.9 (3)

### 5-Ethyl-4-[(4-morpholinobenzylidene)amino]-2,4-dihydro-3H-1,2,4-triazole-3-thione Hydrogen-bond geometry (Å, º)

**Table:**

*D*—H···*A*	*D*—H	H···*A*	*D*···*A*	*D*—H···*A*
N4—H4*N*···S1^i^	0.87 (4)	2.474 (4)	3.335 (2)	179 (14)

Symmetry code: (i) −*x*, −*y*+1, −*z*.
